# Innervation, Motility Peptide, and Extracellular Matrix Remodeling Markers in Proximal and Distal Esophageal Tissue from Children with Esophageal Atresia

**DOI:** 10.3390/medicina62101862

**Published:** 2026-09-25

**Authors:** Veronika Kulineca, Māra Pilmane, Aigars Petersons

**Affiliations:** 1 Faculty of Medicine, Rīga Stradiņš University, 16 Dzirciema Street, LV-1007 Rīga, Latvia; 2 Institute of Anatomy and Anthropology, Rīga Stradiņš University, 16 Dzirciema Street, LV-1007 Rīga, Latvia; 3 Department of Paediatric Surgery, Rīga Stradiņš University, 16 Dzirciema Street, LV-1007 Rīga, Latvia

**Keywords:** esophageal atresia, pediatric esophagus, immunohistochemistry, PGP 9.5, motilin, matrix metalloproteinases, tissue inhibitors of metalloproteinases, extracellular matrix remodeling, proximal and distal segments

## Abstract

*Background and Objectives*: Esophageal atresia (EA) is associated with dysmotility and postoperative morbidity, which may reflect congenital abnormalities of innervation and extracellular matrix (ECM) remodeling. This study compared immunoreactivity for protein gene product 9.5 (PGP 9.5), motilin, three matrix metalloproteinases (MMP-1, MMP-2, and MMP-9), and three tissue inhibitors of metalloproteinases (TIMP-1, TIMP-2, and TIMP-4) among proximal EA, distal EA, and control esophageal tissues. *Materials and Methods*: Twenty-two formalin-fixed, paraffin-embedded EA tissue specimens (10 proximal and 12 distal segments) and five control specimens were evaluated using semiquantitative immunohistochemistry. Nonparametric methods were used for group comparisons, and associations were assessed using Spearman rank correlations. *Results*: Epithelial MMP-1 differed among the groups (*p* = 0.043) and was lower in proximal EA than in controls (pairwise *p* = 0.036). MMP-2 differed in the epithelium (*p* = 0.019) and connective tissue (*p* = 0.022); controls had higher values in the pairwise comparisons. Connective-tissue TIMP-2 also differed among the groups (*p* = 0.021) and was lower in proximal EA than in controls (pairwise *p* = 0.0017). PGP 9.5, MMP-9, TIMP-1, and TIMP-4 showed no significant differences in arithmetic mean values among the groups. Motilin’s immunoreactivity was more pronounced in proximal EA, but epithelial immunoreactivity did not differ significantly among the groups. Exploratory, unadjusted correlations among neural, motility-related, and ECM-remodeling markers were observed in EA tissue. *Conclusions*: The reported differences were specific to the segment and tissue compartment. MMP-2 was lower in distal EA epithelium and in connective tissue from both EA segments than in controls, whereas connective-tissue TIMP-2 was lower in proximal EA. Motilin did not differ significantly among the groups. The correlation findings require confirmation in analyses that adjust for multiple testing.

## 1. Introduction

Esophageal atresia (EA) is a congenital interruption of the esophageal lumen. Although survival after repair has improved, dysphagia, gastroesophageal reflux, aspiration, feeding problems, and dysmotility remain common [1,2,3,4,5,6,7,8,9]. These outcomes may reflect intrinsic abnormalities of the esophageal wall as well as postoperative factors [6,7,8,10,11,12,13,14,15,16,17,18,19,20,21,22,23,24,25,26,27,28,29].

Previous research on human tissue, including the study by Pilmane et al. [20], compared proximal and distal EA segments and evaluated PGP 9.5, MMP-2, growth factors, and their receptors. However, it remains unclear whether motility-related and MMP-inhibitory markers show segment-specific patterns when evaluated alongside neural and matrix-remodeling markers. We therefore evaluated motilin, MMP-1, MMP-9, TIMP-1, TIMP-2, and TIMP-4 in addition to PGP 9.5 and MMP-2. The original contribution of the present study is the combined, compartment-specific assessment of these additional markers within the same proximal, distal, and control-tissue framework; the established proximal-distal comparison itself is not presented as novel. This broader panel provides an original, hypothesis-generating description of motility-related, neural or neuroendocrine, and matrix-remodeling immunoreactivity within one compartment-resolved analysis.

PGP 9.5. Protein gene product 9.5 (PGP 9.5), also known as ubiquitin C-terminal hydrolase-L1, is used to identify neural and neuroendocrine structures [30,31,32,33]. Immunoreactivity was recorded separately in epithelial profiles and nerve tissue. Epithelial positivity was interpreted as marker immunoreactivity rather than direct evidence of epithelial innervation or a specific epithelial cell lineage. A neuroendocrine-like phenotypic interpretation remains a hypothesis that requires cell-type confirmation.

Motilin. Motilin is a 22-amino-acid peptide produced primarily by enteroendocrine cells of the proximal small intestine [34,35]. Motilin receptor signaling influences gastrointestinal motility, and motilin agonists can increase lower esophageal sphincter pressure and esophageal contractility [36,37,38]. Because motilin-positive cells are unexpected in esophageal tissue, their presence in this exploratory immunohistochemical study requires cautious interpretation and independent confirmation. The present study does not establish that staining represents persistence or re-expression of a fetal esophageal cell population.

MMPs and TIMPs. Matrix metalloproteinases and their tissue inhibitors regulate extracellular-matrix turnover and repair [39,40,41,42,43,44]. MMP-1, MMP-2, and MMP-9 act on different matrix substrates, whereas TIMP-1, TIMP-2, and TIMP-4 regulate metalloproteinase activity. Earlier research on EA tissue reported fewer MMP-2-positive structures in the proximal segment [20]; evaluating additional MMPs and TIMPs may clarify whether the differences are specific to a segment or tissue compartment.

Accordingly, this study aimed to compare immunoreactivity for PGP 9.5, motilin, MMP-1, MMP-2, MMP-9, TIMP-1, TIMP-2, and TIMP-4 among proximal EA, distal EA, and control esophageal tissue and to explore compartment-specific correlations among these markers.

## 2. Materials and Methods

The Research Ethics Committee of Rīga Stradiņš University approved the study on 3 March 2026 (decision No. 2-PĒK-4/348/2026).

### 2.1. Selection Criteria for EA Tissue Specimens

The inclusion criteria were:

• Diagnosis of esophageal atresia;
• Both sexes;
• Absence of other congenital diseases;
• Absence of additional pathologies;
• No macroscopically visible inflammation at specimen selection;
• Indication for surgical anastomosis.

The exclusion criteria were:

• Multiple congenital diseases;
• Additional pathology contraindicating reconstructive surgery;
• Macroscopically visible signs of inflammation at specimen selection [45,46,47,48,49].

### 2.2. Characteristics of the EA Tissue Specimens

Table 1 reports every demographic and specimen variable contained in the available archive. The dataset included 22 EA tissue specimens from newborns and infants aged 1 day to 11 months: 10 proximal and 12 distal specimens. Twelve specimens were obtained from male and ten from female patients. Each specimen was obtained from a different patient, and no paired proximal and distal specimens were available; therefore, each patient contributed one specimen only. For clarity, the composite laboratory codes used during processing have been replaced by sequential specimen numbers (Specimen No. 1–22); these numbers are study identifiers and do not correspond to patient identifiers. Patient-level identifiers are not disclosed for ethical reasons. EA type, gestational age, birth weight, operative approach, exact anatomical origin of the tissue, and the prior procedures of the 11-month-old case were not available and were not reconstructed by inference. The study was designed as a basic histo-morphological and histopathogenetic investigation of archived tissue rather than a clinical outcome study; the missing clinical variables remain relevant to interpretation but were not objectives of the present analysis.

### 2.3. Selection and Characteristics of Control Specimens

The control specimens met the following criteria:

• No esophageal atresia documented on examination, history, or family history;
• Postmortem esophageal tissue obtained from cases in which the cause of death was unrelated to EA.

Five control esophageal tissue specimens were obtained during postmortem examination at the Institute of Anatomy and Anthropology, Rīga Stradiņš University. The control group included three female and two male cases, ranging in age from newborn to 1 month. The group was clinically heterogeneous: two cases died of pneumonia, one had Down syndrome with Fallot triad, and one had an unspecified congenital anomaly (Table 2). This small retrospective postmortem series reflects the ethical and practical constraints of obtaining esophageal tissue from healthy children. Parental permission alone would not justify collecting esophageal tissue from otherwise healthy children solely to enlarge a research control group, and the age and diagnostic imbalance could not be eliminated within the available archive.

### 2.4. Routine Staining

Tissue specimens were fixed for 24 h in 2% formaldehyde, 0.2% picric acid, and 0.1 M phosphate buffer (pH 7.2) and then processed for 12 h in Tyrode buffer containing 10% sucrose. They were embedded in paraffin, sectioned at 5–7 µm, and stained with hematoxylin and eosin.

### 2.5. Immunohistochemical Analysis

PGP 9.5, motilin, TIMP-1, TIMP-2, TIMP-4, MMP-1, MMP-2, and MMP-9 were detected immunohistochemically in the selected tissue specimens using the streptavidin-biotin method described below.

Primary antibodies were diluted in antibody diluent (code 938B-05, Cell Marque™, Rocklin, CA, USA). Paraffin sections were deparaffinized and rehydrated through alcohol and water, after which the slides were rinsed twice for 5 min in TRIS buffer solution (code 2017X12508, Diapath S.p.A., Martinengo, Italy). For antigen retrieval, the sections were heated in boiling EDTA buffer (code 2017X02239, Diapath S.p.A., Martinengo, Italy) in a microwave for 20 min and then cooled. The sections were washed twice for 5 min in TRIS buffer, and endogenous peroxidase activity was blocked with 3% peroxide for 10 min, followed by a further TRIS buffer rinse.

Sections were incubated with the primary antibodies for 1 h. They were then washed three times in TRIS buffer and treated with HiDef Detection™ Amplifier (code 954D-31, Cell Marque™, Rocklin, CA, USA) for 10 min at room temperature. After another TRIS buffer wash, the slides were incubated with HiDef Detection™ HRP Polymer Detector (code 954D-32, Cell Marque™, Rocklin, CA, USA) for 10 min at room temperature and then washed three additional times in TRIS buffer for 5 min each.

Immunoreactivity was visualized with a DAB+ chromogenic substrate (DAB Substrate Kit, code 957D-60, Cell Marque™, Rocklin, CA, USA) applied for 10 min. The sections were rinsed under running water and counterstained with hematoxylin (code 05-M06002, Mayer’s Hematoxylin, Bio Optica Milano S.p.A., Milano, Italy). The slides were dehydrated in increasing ethanol concentrations (70–90%), clarified, sealed with coverslips, and coded by specimen identifier and antibody. Table 3 summarizes the product code, working dilution, manufacturer, and location for each primary antibody.

Positive and negative controls were included in every staining run. Positive controls were the tissue sections indicated by the antibody manufacturers, which showed the expected immunoreactivity; negative controls were processed identically with the primary antibody omitted. For motilin, a small-intestinal (duodenal) section served as the motilin-specific positive control, because motilin-producing enteroendocrine cells are normally present in the duodenal epithelium [50,51,52,53,54,55,56]. Motilin cells have been identified in the duodenal epithelium of premature infants [54] and in the human fetal gastro-entero-pancreatic system [55,56].

### 2.6. Semiquantitative Assessment of Immunoreactivity

Light microscopy and semiquantitative counting were used to assess the relative quantity of structures positive for PGP 9.5, motilin, TIMP-1, TIMP-2, TIMP-4, MMP-1, MMP-2, and MMP-9 in the mucosal epithelium, connective tissue, blood vessels, and nerve tissue, as applicable. Positively stained structures in each visual field were classified using the categories in Table 4. The percentages in Table 4 represent the estimated proportion of assessed structures in a visual field that showed positive immunoreactivity; 100% indicates that all assessed structures in that field were immunoreactive. In the source scoring sheets, ‘nerve tissue’ was used as a compartment label for putative nerve-associated profiles. The archived protocol did not define separate morphological criteria or neural-marker co-localization for classifying motilin- or TIMP-immunoreactive structures in this compartment. These values therefore represent descriptive semiquantitative scores in source-labeled nerve-associated profiles, not confirmed neuronal expression or cell identity.

Two independent morphologists evaluated the slides without access to patient or group identifiers and were informed only of the antibody being assessed. Each morphologist scored the slides independently, and disagreements were resolved by consensus.

Images were acquired with a Leica DC 300F digital camera (Leica Microsystems Digital Imaging, Cambridge, UK). No image-processing or image-analysis software was used; immunoreactivity was assessed by light microscopy and semiquantitative counting as described above. Group values in Table 5 and Figure 1 are arithmetic means of the individual specimen scores: the individual scores were mapped onto the 0–4 category scale shown in Table 4, and the mean of the available measurements was assigned to the corresponding category.

**Figure 1:**
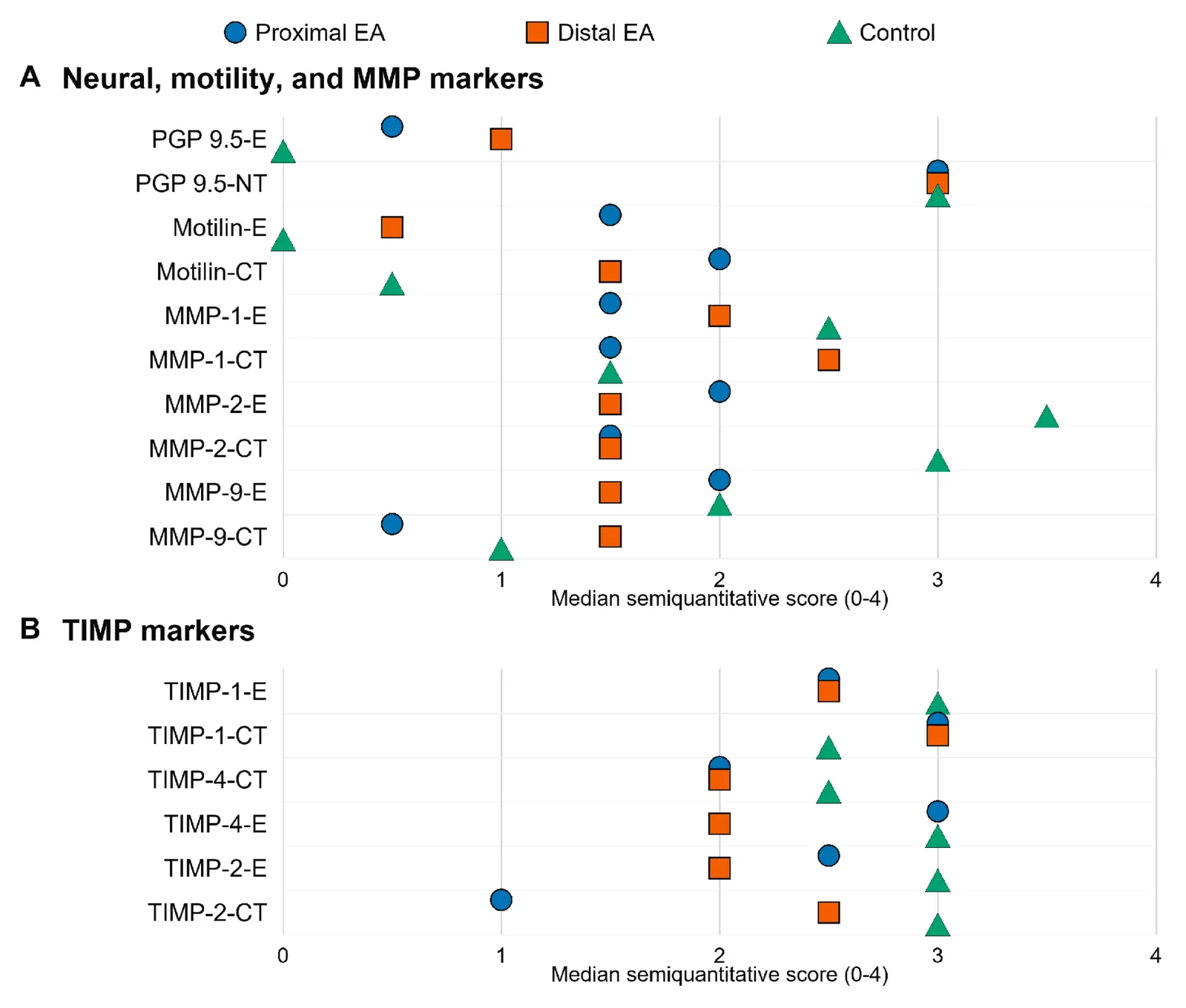
Arithmetic mean semiquantitative scores (0–4) for PGP 9.5, motilin, MMP-1, MMP-2, MMP-9, TIMP-1, TIMP-2, and TIMP-4 in proximal EA, distal EA, and control groups.

### 2.7. Statistical Analysis

Statistical analyses were performed using IBM SPSS Statistics version 26.0 (IBM, Chicago, IL, USA). Because the semiquantitative scores were ordinal, descriptive and nonparametric methods were used. Groups were compared using the Kruskal–Wallis test, which was computed separately for each marker and tissue compartment, and associations were evaluated using Spearman rank correlations. The nominal significance threshold was *p* < 0.05. The correlation analyses were not adjusted for multiple testing and were therefore considered exploratory. Spearman correlation strength was classified as very weak (|rho| = 0.00–0.19), weak (0.20–0.39), moderate (0.40–0.59), strong (0.60–0.79), or very strong (0.80–1.00) [63].

### 2.8. Study Flow

Figure 2 summarizes the steps described in Section 2.

## 3. Results

### 3.1. Histological Findings in H&E-Stained Sections

Although specimens with macroscopically visible inflammation were excluded during selection, microscopic examination of the EA sections showed nonkeratinized stratified squamous epithelium with focal cytoplasmic vacuolization, thickening of the mucosal muscle layer, and subepithelial inflammatory cell infiltration. The lamina propria and submucosal stroma appeared fibrous and irregular (Figure 3a–c).

### 3.2. PGP 9.5 Immunoreactivity

In controls, the arithmetic mean score for epithelial PGP 9.5-positive structures was absent (0), whereas nerve tissue contained numerous (+++) immunopositive structures.

In proximal EA, epithelial PGP 9.5-immunoreactive cells were rare (0/+), whereas nerve structures were numerous (+++). In distal EA, epithelial PGP 9.5-positive structures were few (+), and nerve tissue also contained numerous (+++) PGP 9.5-positive structures (Table 5).

Neither epithelial nor nerve-associated PGP 9.5 immunoreactivity differed significantly among controls, proximal EA, and distal EA (Figure 4a–c).

### 3.3. Motilin Immunoreactivity

In controls, the arithmetic mean score for motilin-positive structures was absent (0) in the epithelium and rare (0/+) in connective tissue.

In proximal EA, the arithmetic mean score for motilin-immunoreactive cells was moderate (+/++) in the epithelium and moderate (++) in connective tissue.

In distal EA, the arithmetic mean motilin score was rare (0/+) in the epithelium and moderate (+/++) in connective tissue (Table 5). Epithelial motilin did not differ significantly among the groups (Figure 5a–c).

### 3.4. MMP-1 Immunoreactivity

In controls, the arithmetic mean score for MMP-1-positive structures was moderate to numerous (++/+++) in the epithelium and few to moderate (+/++) in connective tissue.

In proximal EA, the arithmetic mean MMP-1 score was few to moderate (+/++) in both the epithelium and connective tissue.

In distal EA, the arithmetic mean MMP-1 score was moderate (++) in the epithelium, whereas connective tissue contained numerous (++/+++) MMP-1-positive cells (Figure 6a–c).

Epithelial MMP-1 differed significantly among the groups (Kruskal–Wallis *p* = 0.043). In the pairwise comparison, epithelial MMP-1 was lower in proximal EA than in controls (*p* = 0.036). The connective-tissue differences were not statistically significant (*p* > 0.05) (Table 5).

### 3.5. MMP-2 Immunoreactivity

In controls, the arithmetic mean score for MMP-2-positive structures was numerous to abundant (+++/++++) in the epithelium and numerous (+++) in connective tissue.

In proximal EA, the arithmetic mean MMP-2 score was moderate (++) in the epithelium and few to moderate (+/++) in connective tissue.

In distal EA, the arithmetic mean MMP-2 score was few to moderate (+/++) in both the epithelium and connective tissue (Figure 7a–c; Table 5).

Epithelial and connective-tissue MMP-2 differed significantly among the groups (Kruskal–Wallis: epithelium *p* = 0.019; connective tissue *p* = 0.022). In the pairwise comparisons, epithelial MMP-2 was higher in controls than in distal EA (*p* = 0.015), and connective-tissue MMP-2 was higher in controls than in proximal EA (*p* = 0.049) and distal EA (*p* = 0.025) (Table 5).

### 3.6. MMP-9 Immunoreactivity

In controls, the arithmetic mean MMP-9 score was moderate (++) in the epithelium, whereas the connective-tissue score was few (+).

In proximal EA, the arithmetic mean MMP-9 score was moderate (++) in the epithelium and rare (0/+) in connective tissue.

In distal EA, the arithmetic mean MMP-9 score was few to moderate (+/++) in both the epithelium and connective tissue (Figure 8a–c). Neither epithelial nor connective-tissue MMP-9 differed significantly among the groups (Table 5).

### 3.7. TIMP-1 Immunoreactivity

In controls, the arithmetic mean score for TIMP-1-positive structures was numerous (+++) in the epithelium and moderate to numerous (++/+++) in connective tissue (Table 5).

In both proximal and distal EA, the arithmetic mean TIMP-1 score was numerous (+++) in the epithelium and moderate (++) in connective tissue (Figure 9a–c). Neither epithelial nor connective-tissue TIMP-1 differed significantly among the groups (*p* > 0.05).

### 3.8. TIMP-2 Immunoreactivity

In controls, the arithmetic mean score for TIMP-2-positive structures was numerous (+++) in the epithelium and moderate to numerous (++/+++) in connective tissue.

In proximal EA, the arithmetic mean TIMP-2 score was moderate to numerous (++/+++) in the epithelium but few (+) in connective tissue.

In distal EA, the arithmetic mean TIMP-2 score was moderate (++) in the epithelium and moderate to numerous (++/+++) in connective tissue (Figure 10a–c).

Connective-tissue TIMP-2 differed significantly among the groups (*p* = 0.021). In the pairwise comparison, connective-tissue TIMP-2 was lower in proximal EA than in controls (*p* = 0.0017). Epithelial TIMP-2 did not differ significantly among the groups (*p* > 0.05) (Table 5).

### 3.9. TIMP-4 Immunoreactivity

In controls, the arithmetic mean score for TIMP-4-positive structures was numerous (+++) in connective tissue and moderate to numerous (++/+++) in the epithelium.

In proximal EA, the arithmetic mean TIMP-4 score was moderate (++) in connective tissue and numerous (+++) in the epithelium. In distal EA, the arithmetic mean TIMP-4 score was moderate (++) in both connective tissue and the epithelium (Figure 11a–c).

TIMP-4 did not differ significantly among the groups in either analyzed compartment (Kruskal–Wallis *p* > 0.05) (Table 5).

### 3.10. Comparison of Marker Immunoreactivity

Figure 1 summarizes the arithmetic mean semiquantitative scores for PGP 9.5, motilin, MMP-1, MMP-2, MMP-9, TIMP-1, TIMP-2, and TIMP-4 across proximal EA, distal EA, and control tissue (Table 5).

### 3.11. Correlations in EA Tissue

Spearman rank correlations were used to evaluate associations among innervation (PGP 9.5), motility (motilin), and extracellular-matrix remodeling markers (MMP-1, MMP-2, MMP-9, TIMP-1, TIMP-2, and TIMP-4) in EA tissue. Table 6 presents the resulting correlation-coefficient matrix for all 22 EA specimens. The strongest coefficients are summarized below, and each quoted value corresponds to a cell of Table 6.

The strongest positive coefficients in Table 6 were those between nerve-tissue PGP 9.5 and epithelial PGP 9.5 (rs = 0.674), between nerve-tissue PGP 9.5 and epithelial TIMP-1 (rs = 0.736), between epithelial PGP 9.5 and connective-tissue TIMP-4 (rs = 0.616), between epithelial MMP-2 and epithelial TIMP-4 (rs = 0.637), between connective-tissue MMP-9 and connective-tissue TIMP-4 (rs = 0.624), and between epithelial TIMP-1 and epithelial TIMP-4 (rs = 0.667). All six coefficients were positive.

Moderate coefficients of similar direction were also present. Nerve-tissue PGP 9.5 correlated positively with connective-tissue TIMP-4 (rs = 0.514) and with nerve-tissue TIMP-4 (rs = 0.507); epithelial MMP-2 correlated positively with connective-tissue MMP-2 (rs = 0.544), epithelial TIMP-1 (rs = 0.433), and nerve-tissue TIMP-1 (rs = 0.458); nerve-tissue TIMP-1 correlated positively with connective-tissue MMP-2 (rs = 0.548); and connective-tissue MMP-1 correlated positively with connective-tissue MMP-9 (rs = 0.509). Moderate negative coefficients involved epithelial motilin with nerve-tissue TIMP-1 (rs = −0.520), epithelial motilin with connective-tissue MMP-9 (rs = −0.457), epithelial motilin with connective-tissue TIMP-4 (rs = −0.457), and epithelial MMP-1 with connective-tissue TIMP-1 (rs = −0.517). The remaining coefficients involving motilin were weak (|rs| < 0.40).

## 4. Discussion

Histological findings in EA tissue included vacuolized squamous epithelium, thickening of the mucosal muscle layer, and subepithelial inflammatory cell infiltration. Immunohistochemical findings varied by segment and tissue compartment. The pairwise comparisons showed lower epithelial MMP-1 in proximal EA, lower epithelial MMP-2 in distal EA, lower connective-tissue MMP-2 in both EA segments, and lower connective-tissue TIMP-2 in proximal EA than in controls. Epithelial motilin immunoreactivity did not differ significantly among the groups (*p* = 0.060).

Nerve-associated PGP 9.5 immunoreactivity did not differ significantly among the groups; nerve structures remained numerous in all groups, whereas epithelial PGP 9.5 immunoreactivity was low. Because PGP 9.5 is used as a neural and neuroendocrine marker, epithelial immunoreactivity alone neither identifies the stained cells nor establishes epithelial innervation. The unadjusted correlations are exploratory and do not demonstrate altered neuroepithelial–stromal coupling.

Motilin immunoreactivity was descriptively more pronounced in proximal EA, whereas the epithelial group comparison was not statistically significant (*p* = 0.060). Motilin is produced primarily by enteroendocrine cells of the proximal small intestine [13,14], making esophageal staining unexpected. Disturbed foregut development in EA may permit focal ectopic neuroendocrine-like differentiation [22,23]. The proximal staining pattern could also be consistent with compensatory activation of local motility signaling in the developmentally abnormal pouch. This remains a hypothesis: immunoreactivity alone does not establish cell identity, peptide release, or a functional motility response.

MMP-2 findings were compartment-specific. Epithelial MMP-2 was lower in distal EA than in controls, whereas connective-tissue MMP-2 was lower in both proximal and distal EA. No significant proximal epithelial difference was observed. MMP-2 was therefore not uniformly reduced across EA tissue compartments. Earlier work reported fewer MMP-2-positive structures in the proximal atretic segment [20]; comparison with that study must account for the tissue compartment and scoring approach. The present series shares no patients or tissue specimens with the earlier study by Pilmane et al. [20]; the specimens are therefore unique, and the present data can be regarded as a continuation of that work.

By contrast, connective-tissue MMP-1 was relatively more pronounced in distal EA, whereas epithelial MMP-1 was lower in proximal EA than in controls. Because MMP-1 participates in fibrillar collagen degradation and epithelial migration [39,40,41,42,43,44,64,65,66], the proximal decrease may reflect impaired reparative turnover, whereas distal connective-tissue reactivity may be secondary to chronic exposure to fistula-associated luminal content, reflux, or other postnatal injury-related stimuli. Thus, proximal and distal EA tissue should not be interpreted as biologically identical compartments; the distal segment appears more reactive, whereas the proximal pouch may be more profoundly developmentally altered.

MMP-9, TIMP-1, and TIMP-4 showed no statistically significant differences in arithmetic mean values among the groups. Several strong nominal correlations were observed, but more than 150 coefficients were tested per segment without correction for multiple comparisons. These patterns are hypothesis-generating and cannot be interpreted as coordinated biological regulation without confirmation in analyses adjusted for multiple comparisons and in an independent cohort.

Experimental studies outside the esophagus show that TIMP-1 can be expressed by neurons and astrocytes, TIMP-2 is associated with neural differentiation, and TIMP-4 is strongly expressed in brain tissue [67,68,69]. These findings provide biological context for nerve-associated immunoreactivity but do not establish neuronal localization in EA esophagus.

Connective-tissue TIMP-2 was lower in proximal EA than in controls. TIMP-2 can participate in both the activation and inhibition of MMP-2, depending on local conditions [41,42,43,44,65,70,71]. The concurrent findings in proximal connective tissue are compatible with an altered MMP-2/TIMP-2 axis, but semiquantitative immunohistochemistry cannot establish functional activity or mechanism. Correlations with PGP 9.5 and other markers remain exploratory until the analyses are corrected for multiple testing.

Overall, the results describe segment- and compartment-specific differences in selected remodeling markers. The small and clinically heterogeneous control group, the semiquantitative design, and the uncorrected correlation analysis prevent firm conclusions about mechanisms of dysmotility or postoperative complications.

### Study Limitations

This study has several limitations. It included 22 EA specimens and five controls. The 22 EA specimens were obtained from 22 different patients, with one specimen per patient and no paired proximal and distal specimens; patient-level identifiers are not disclosed for ethical reasons. EA type, gestational age, birth weight, operative approach, exact tissue origin, and the prior procedures of the 11-month-old case were unavailable. This limitation indicates a direction for future research, namely comparison of the morphological findings with clinical data. The small, unequal control group was clinically heterogeneous: two controls died of pneumonia, one had Down syndrome with Fallot triad, one had an unspecified congenital anomaly, and ages were not fully comparable with those of the EA specimens. Ethical and practical constraints precluded the collection of esophageal tissue from healthy children. These constraints do not eliminate selection bias or confounding, and the age and diagnostic imbalance could not be removed within the retrospective archive. Additionally, the cross-sectional design cannot determine how long MMP/TIMP remodeling patterns require to develop.

Semiquantitative immunohistochemistry is observer-dependent and does not provide absolute protein concentrations; thus, independent confirmation by ELISA, radioimmunoassay, or Western blotting would be a valuable direction for future research. Although slide evaluation was masked to patient and group identifiers and disagreements were resolved by consensus, no quantitative statistic of interobserver agreement was available.

More than 150 correlations were tested per segment without correction for multiple comparisons, increasing the risk of false-positive findings; the correlations are therefore exploratory. The cross-sectional design cannot distinguish developmental abnormalities from the effects of fistula exposure, reflux, inflammation, or clinical management. These limitations require cautious interpretation.

## 5. Conclusions

The study identified the following exploratory, segment- and compartment-specific differences in selected neural, motility-related, and extracellular-matrix markers:

1. Numerous nerve-associated PGP 9.5-positive structures in all research groups indicate preserved neural elements, whereas distal correlation patterns seem more suggesting of some tissue dysmotility in EA.
2. Motilin immunoreactivity was descriptively more pronounced in proximal EA, although epithelial motilin immunoreactivity did not differ significantly among the groups, suggesting that compensatory activation of local motility signaling occurred in the developmentally abnormal proximal pouch.
3. Diminished MMP-1 immunoreactivity in the proximal EA epithelium and decreased MMP-2 immunoreactivity in distal EA epithelium and in connective tissue from both EA segments may indicate altered extracellular matrix remodeling associated with EA. Decreased TIMP-2 immunoreactivity in the connective tissue of proximal EA further suggests weakened extracellular matrix homeostasis in the proximal pouch.
4. MMP-9, TIMP-1, and TIMP-4 showed no significant differences in arithmetic mean values among the groups and are therefore less likely to represent major postnatal pathogenetic factors in EA.

## Figures and Tables

**Figure 2:**
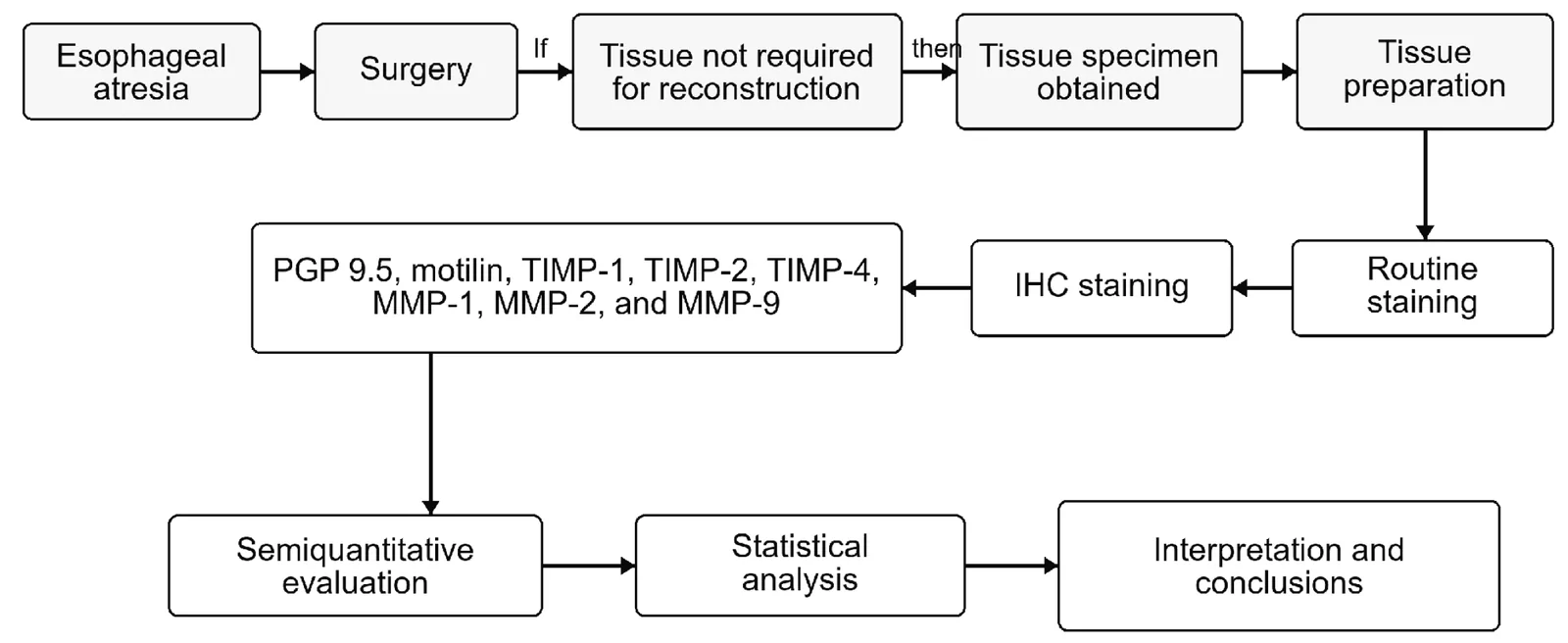
Flowchart of sample selection and processing.

**Figure 3:**
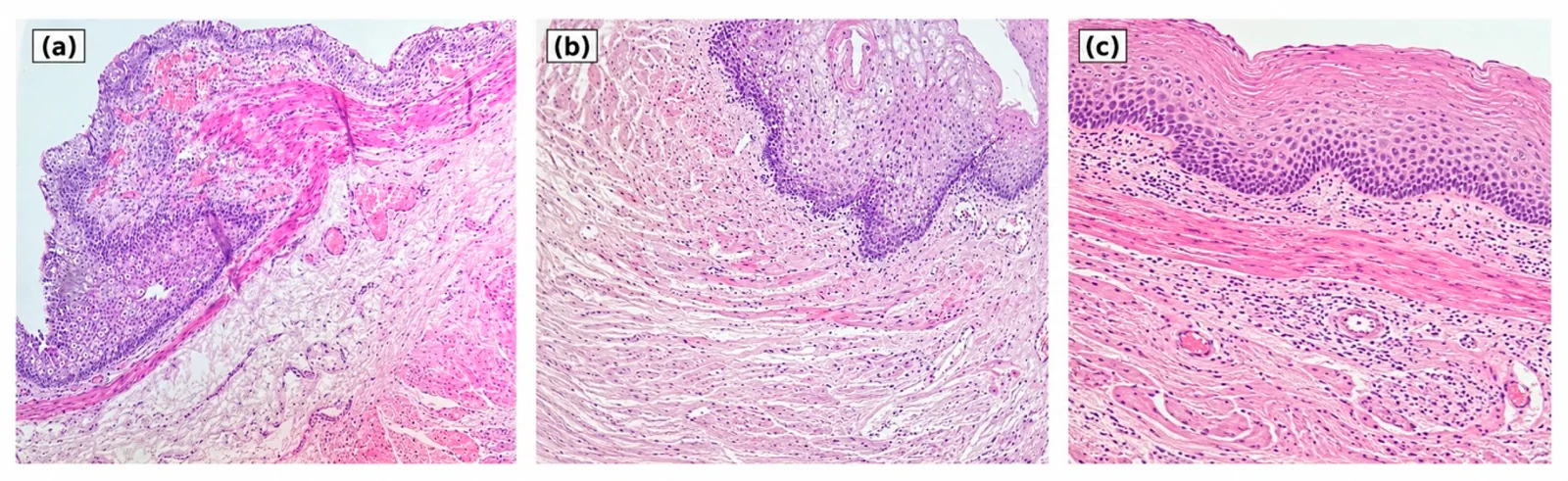
H&E findings in proximal EA (**a**), distal EA (**b**), and control (**c**) tissue. Compared with control squamous epithelium and mucosal muscle, the EA panels show vacuolated stratified squamous epithelium, microscopic subepithelial inflammatory infiltration, and a thickened mucosal muscle layer.

**Figure 4:**
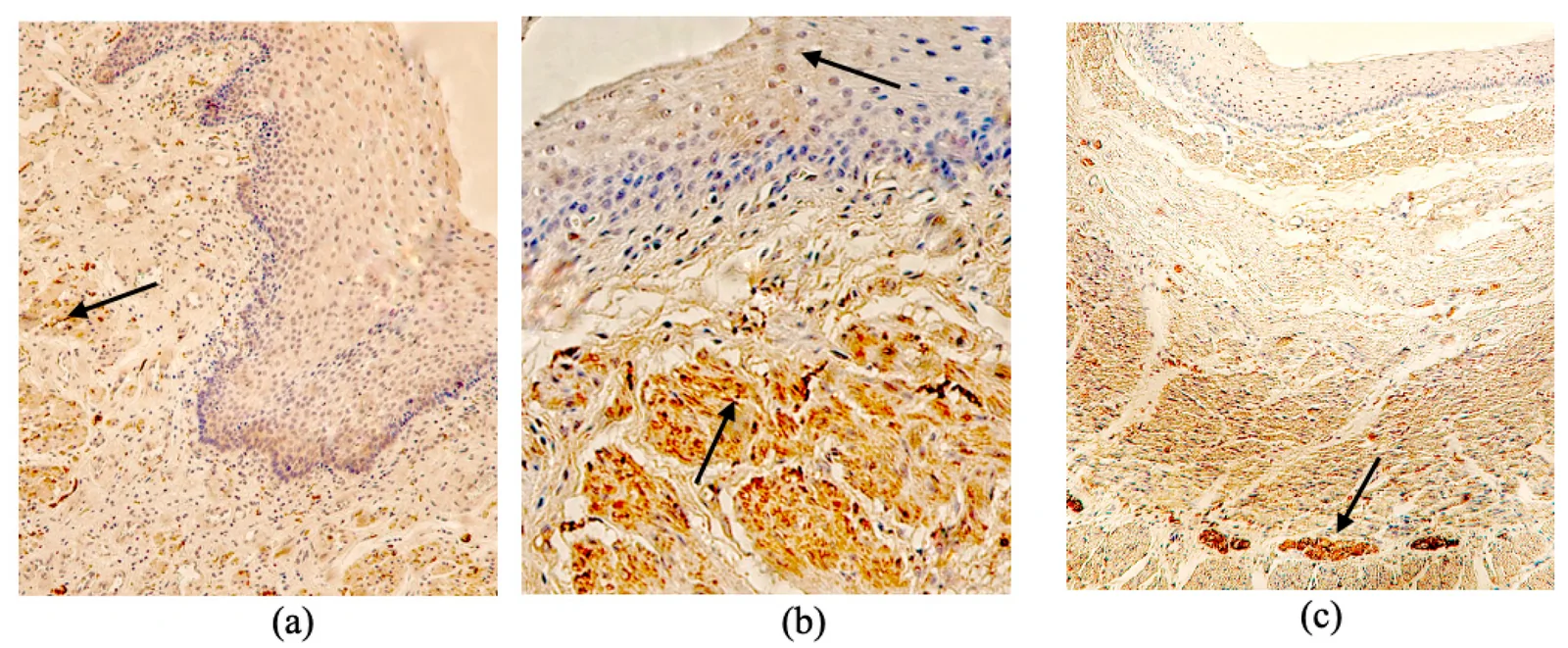
PGP 9.5 immunohistochemistry in proximal EA (**a**), distal EA (**b**), and control (**c**) tissue. Arrows indicate PGP 9.5-positive epithelial or nerve structures; original magnification, 100×.

**Figure 5:**
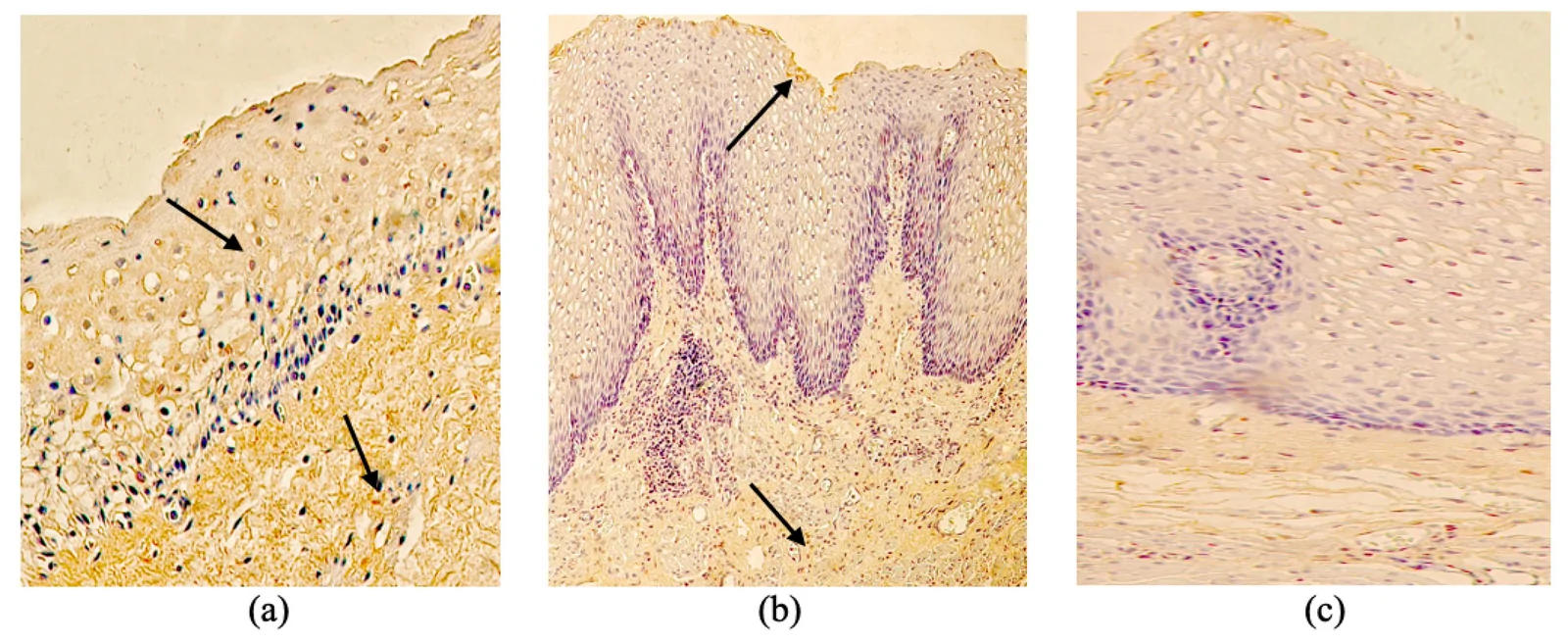
Motilin immunohistochemistry in proximal EA (**a**), distal EA (**b**), and control (**c**) tissue. Arrows indicate motilin-containing cells; original magnification, 100×.

**Figure 6:**
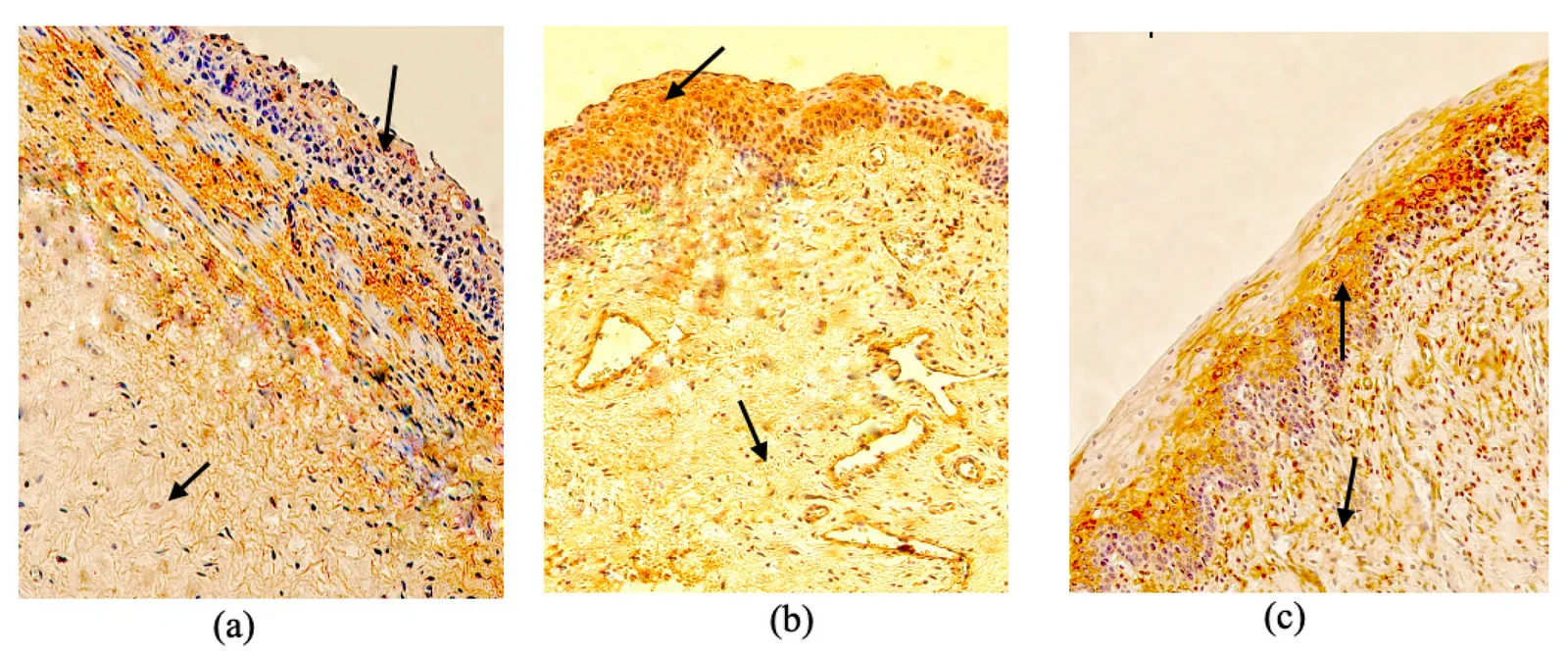
MMP-1 immunohistochemistry in proximal EA (**a**), distal EA (**b**), and control (**c**) tissue. Arrows indicate MMP-1-containing epithelial and connective tissue cells; original magnification, 100×.

**Figure 7:**
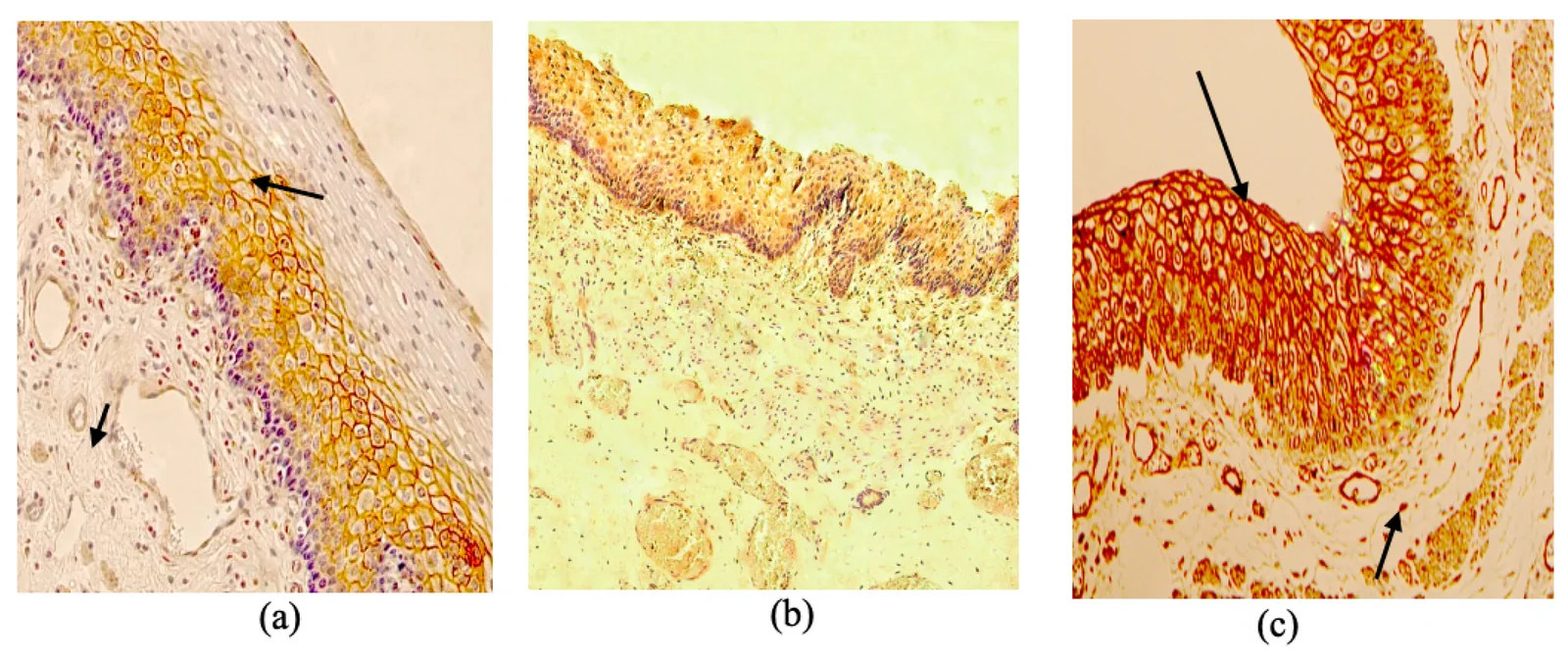
MMP-2 immunohistochemistry in proximal EA (**a**), distal EA (**b**), and control (**c**) tissue. Arrows indicate MMP-2-containing epithelial and connective tissue cells; original magnification, 100×.

**Figure 8:**
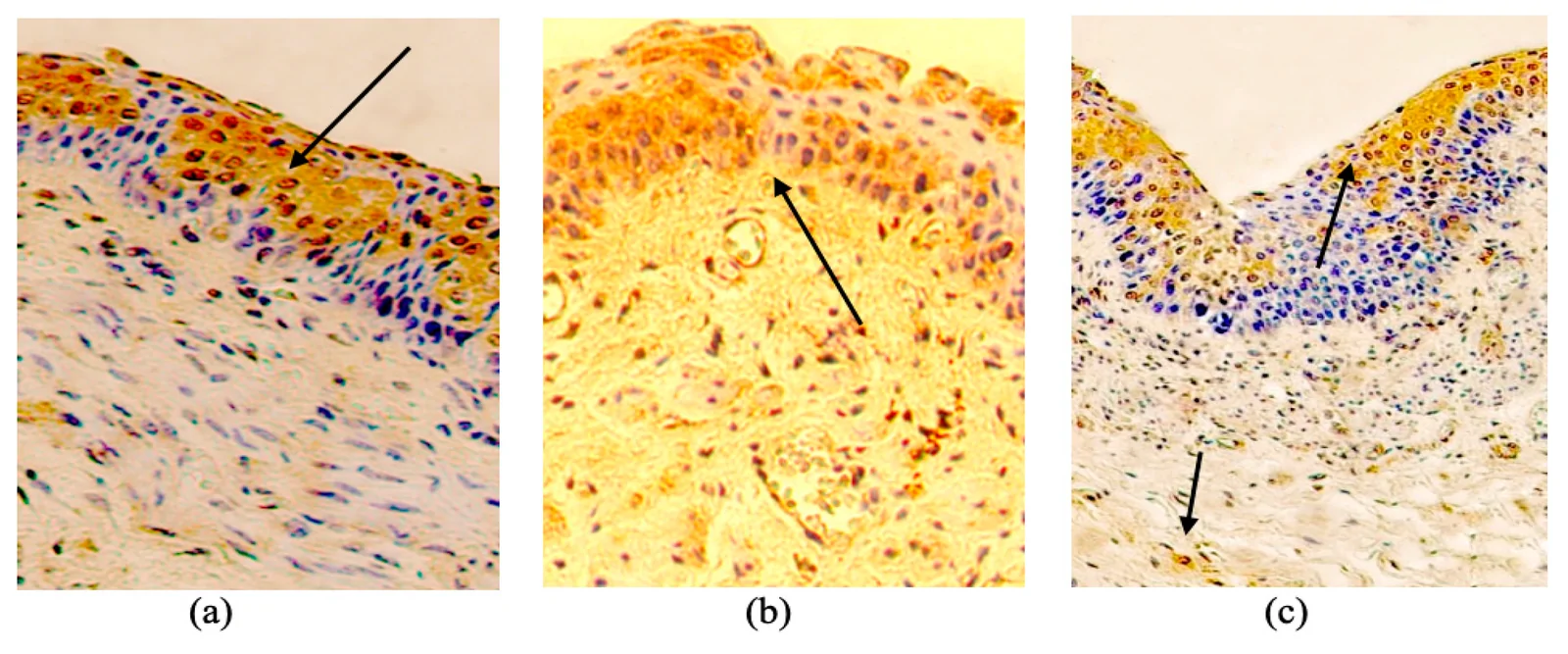
MMP-9 immunohistochemistry in proximal EA (**a**), distal EA (**b**), and control (**c**) tissue. Arrows indicate MMP-9-containing epithelial and connective tissue cells; original magnification, 100×.

**Figure 9:**
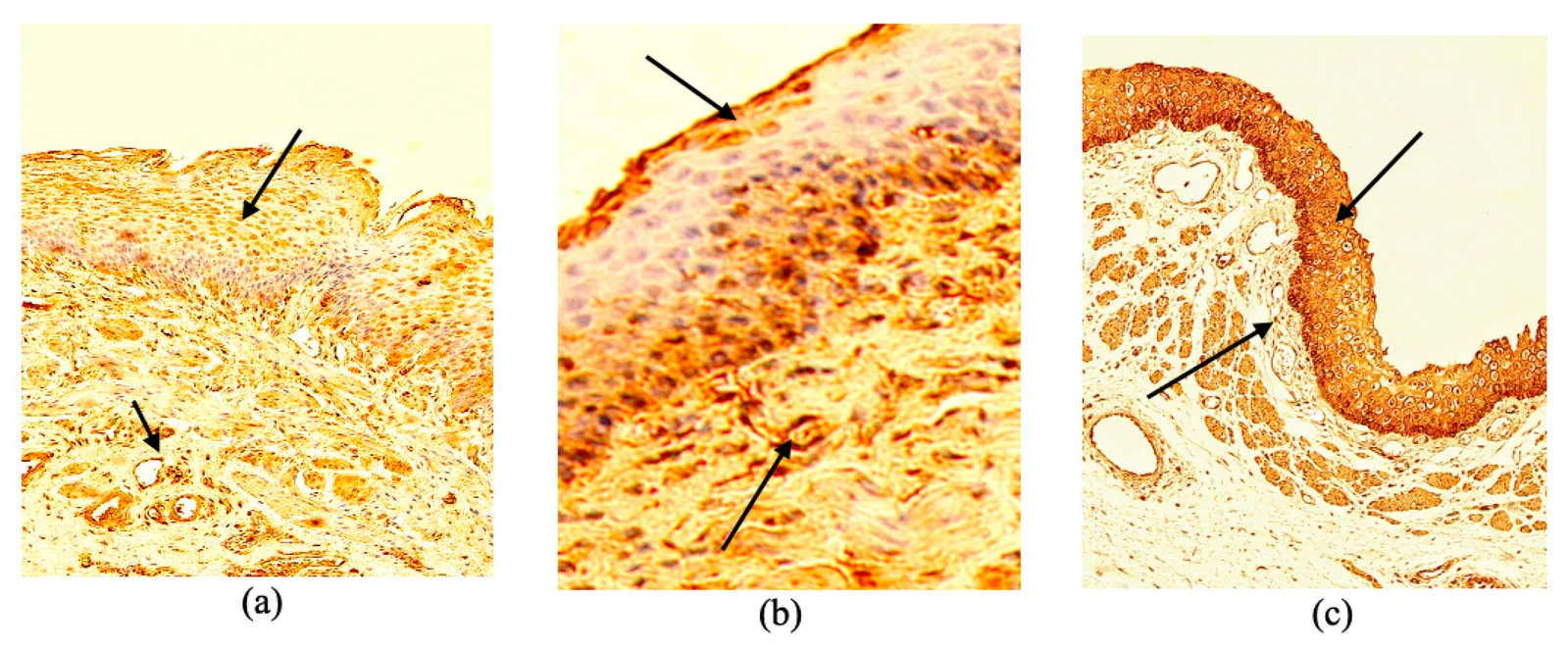
TIMP-1 immunohistochemistry in proximal EA (**a**), distal EA (**b**), and control (**c**) tissue. Arrows indicate TIMP-1-positive epithelial and connective tissue structures; original magnification, 100×.

**Figure 10:**
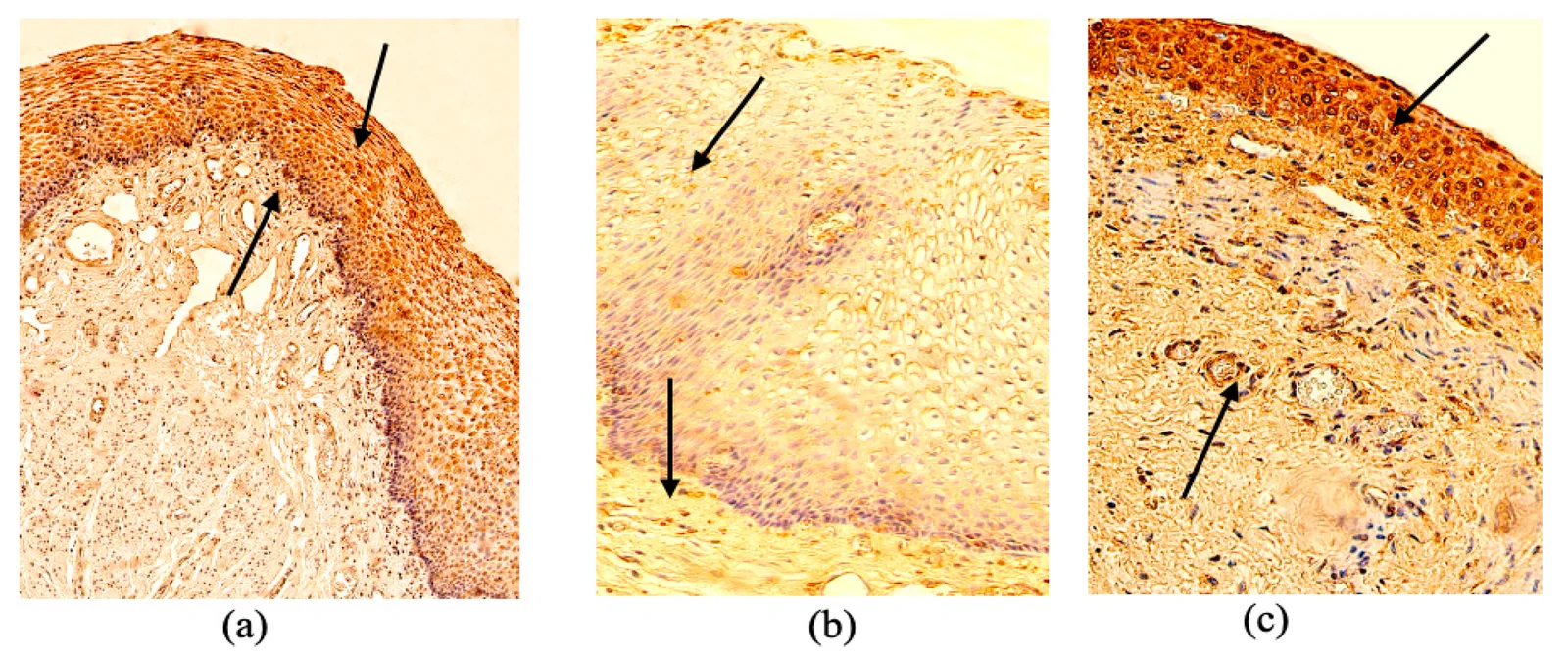
TIMP-2 immunohistochemistry in proximal EA (**a**), distal EA (**b**), and control (**c**) tissue. Arrows indicate TIMP-2-positive epithelial and connective tissue structures; original magnification, 100×.

**Figure 11:**
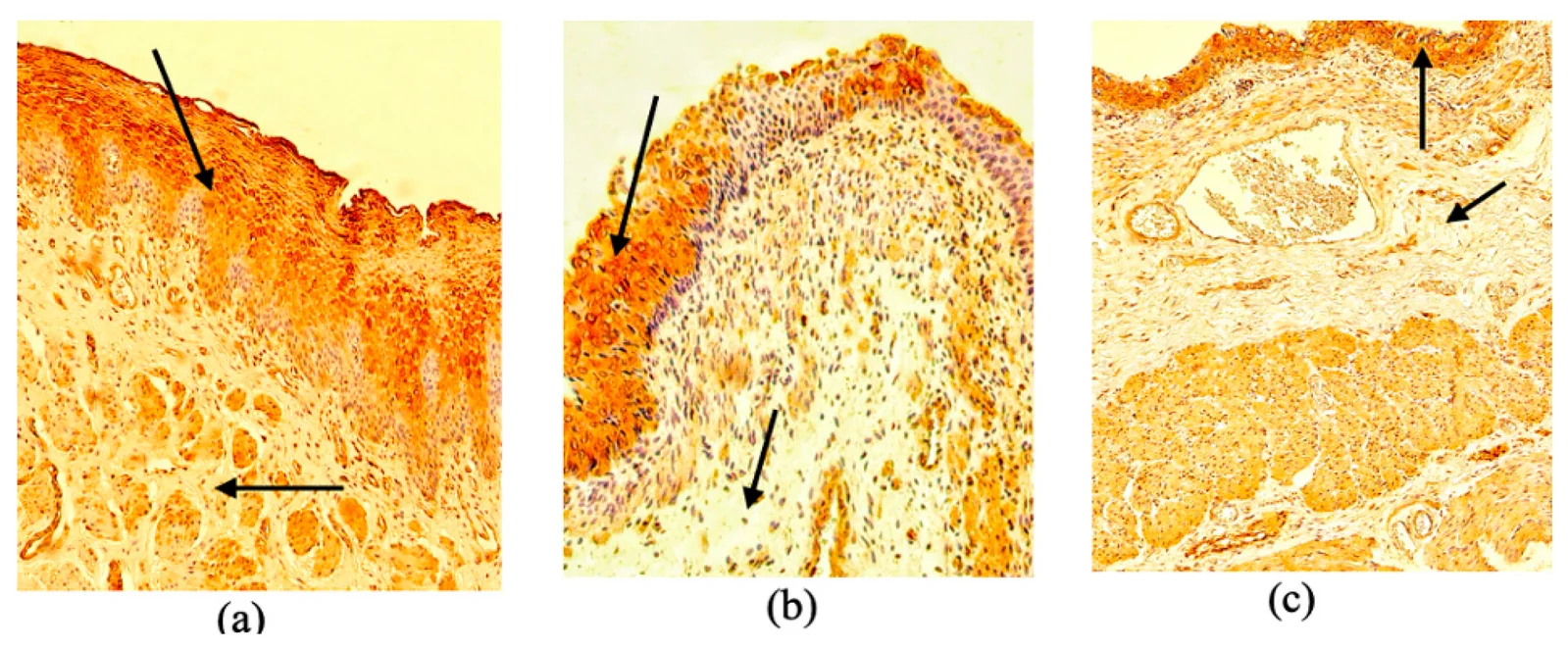
TIMP-4 immunohistochemistry in proximal EA (**a**), distal EA (**b**), and control (**c**) tissue. Arrows indicate TIMP-4-positive epithelial and connective tissue structures; original magnification, 100×.

**Table 1:** Characteristics of the EA tissue specimens.

Specimen No.	Sex	Age	Diagnosis	Segment
1	M	1 d	Atresia esophagi	Prox.
2	F	1 d	Atresia esophagi	Prox.
3	M	1 d	Atresia esophagi	Dist.
4	F	1 d	Atresia esophagi	Dist.
5	M	1 d	Atresia esophagi	Prox.
6	F	1 d	Atresia esophagi	Dist.
7	F	1 d	Atresia esophagi	Prox.
8	F	1 d	Atresia esophagi	Dist.
9	M	1 d	Atresia esophagi	Dist.
10	M	1 d	Atresia esophagi	Prox.
11	F	1 d	Atresia esophagi	Dist.
12	M	3 d	Atresia esophagi	Dist.
13	F	4 d	Atresia esophagi	Dist.
14	F	4 d	Atresia esophagi	Dist.
15	M	4 d	Atresia esophagi	Dist.
16	M	4 d	Atresia esophagi	Prox.
17	F	21 d	Atresia esophagi	Dist.
18	M	1 m	Atresia esophagi	Prox.
19	M	4 m	Atresia esophagi	Dist.
20	M	11 m	Atresia esophagi	Prox.
21	M	11 m	Atresia esophagi	Prox.
22	F	3 d	Atresia esophagi	Prox.

Abbreviations: M, male; F, female; Prox., proximal esophagus; Dist., distal esophagus; d, day; m, month.

**Table 2:** Characteristics of the control group.

Control	Age	Sex	Cause of Death
1.K	1 m	F	Bilateral pneumonia
2.K	1 d	F	Congenital anomaly/birth defect
3.K	1 d	F	Fallot triad with Down syndrome
4.K	1 m	M	Necrotizing pneumonia
5.K	1 d	M	Sudden death syndrome

Abbreviations: M, male; F, female; d, day; m, month.

**Table 3:** Primary antibodies used for immunohistochemistry.

Target	Product Code	Dilution	Manufacturer	Location
PGP 9.5	ab8189	1:100	Abcam	Cambridge, UK
Motilin	b23-100	1:10	Dako	Copenhagen, Denmark
TIMP-1	orb195994_1	1:400	Biorbyt	Cambridge, UK
TIMP-2	sc-21735	1:50	Santa Cruz Biotechnology, Inc.	Santa Cruz, CA, USA
TIMP-4	orb106543	1:100	Biorbyt	Cambridge, UK
MMP-1	sc-21731	1:50	Santa Cruz Biotechnology, Inc.	Santa Cruz, CA, USA
MMP-2	orb101049	1:100	Biorbyt	Cambridge, UK
MMP-9	Ab76003	1:100	Abcam	Cambridge, UK

**Table 4:** Categories used for semiquantitative evaluation [57,58,59,60,61]. Percentages represent the estimated proportion of assessed structures in a visual field that showed positive immunoreactivity. Reprinted from Rone et al. (2026) [62].

Identifier	Interpretation
0	No positive structures in the visual field (0%)
0/+	Rare positive structures (12.5%)
+	Few positive structures (25%)
+/++	Few to moderate positive structures (37.5%)
++	Moderate positive structures (50%)
++/+++	Moderate to numerous positive structures (62.5%)
+++	Numerous positive structures (75%)
+++/++++	Numerous to abundant positive structures (87.5%)
++++	Abundant positive structures (100%)

**Table 5:** Arithmetic mean semiquantitative scores for proximal EA, distal EA, and control specimens, with Kruskal–Wallis *p*-values. The arithmetic mean is calculated using data from Appendix A and Appendix B.

(**A**) Neural, motility, and MMP markers																
**Part**	**PGP 9.5-E**		**PGP 9.5-NT**	**Motilin-E**		**Motilin-CT**		**MMP-1-E**	**MMP-1-CT**		**MMP-2-E**		**MMP-2-CT**	**MMP-9-E**		**MMP-9-CT**
Prox.	0/+		+++	+/++		++		+/++	+/++		++		+/++	++		0/+
Dist.	+		+++	0/+		+/++		++	++/+++		+/++		+/++	+/++		+/++
Control	0		+++	0		0/+		++/+++	+/++		+++/++++		+++	++		+
p	0.396		0.696	0.060		0.134		0.043	0.055		0.019		0.022	0.627		0.165
(**B**) TIMP markers																
**Part**		**TIMP-1-E**			**TIMP-1-CT**		**TIMP-4-CT**			**TIMP-4-E**		**TIMP-2-E**			**TIMP-2-CT**	
Prox.		++/+++			+++		++			+++		++/+++			+	
Dist.		++/+++			+++		++			++		++			++/+++	
Control		+++			++/+++		++/+++			+++		+++			+++	
p		0.401			0.207		0.790			0.243		0.245			0.021	

Abbreviations: E, epithelium; CT, connective tissue; NT, nerve tissue; MMP, matrix metalloproteinase; TIMP, tissue inhibitor of metalloproteinases; Prox., proximal segment; Dist., distal segment.

**Table 6:** Spearman correlation-coefficient matrix for all 22 EA specimens.

Variable	PGP 9.5-E	PGP 9.5-NT	Motilin-E	Motilin-CT	MMP-1-E	MMP-1-CT	MMP-2-E	MMP-2-CT
(**A**) First eight coefficient columns								
PGP 9.5-E	--							
PGP 9.5-NT	0.674	--						
Motilin-E	−0.306	−0.063	--					
Motilin-CT	0.215	0.082	−0.065	--				
MMP-1-E	−0.391	−0.404	0.021	−0.356	--			
MMP-1-CT	0.127	−0.064	−0.153	0.339	−0.007	--		
MMP-2-E	−0.042	−0.152	−0.187	−0.389	0.383	−0.162	--	
MMP-2-CT	0.045	0.206	−0.101	−0.179	0.209	−0.160	0.544	--
MMP-9-E	0.106	0.143	−0.011	0.122	0.140	0.101	0.352	0.176
MMP-9-CT	0.226	0.047	−0.457	0.300	0.049	0.509	0.391	0.066
TIMP-1-E	0.217	0.736	−0.260	−0.193	−0.129	−0.277	0.433	0.540
TIMP-1-CT	0.233	0.167	−0.029	0.125	−0.517	0.269	0.191	0.067
TIMP-1-NT	0.089	−0.061	−0.520	−0.258	0.429	0.139	0.458	0.548
TIMP-4-CT	0.616	0.514	−0.457	0.296	−0.138	0.281	0.251	0.293
TIMP-4-E	0.247	0.406	−0.240	−0.370	−0.111	−0.230	0.637	0.461
TIMP-4-NT	0.433	0.507	−0.172	0.197	−0.471	0.347	−0.064	−0.087
TIMP-2-E	−0.008	0.216	−0.026	−0.330	0.033	−0.446	0.288	0.455
(**B**) Remaining coefficient columns								
**Variable**	**MMP-9-E**	**MMP-9-CT**	**TIMP-1-E**	**TIMP-1-CT**	**TIMP-4-CT**	**TIMP-4-E**	**TIMP-2-E**	**TIMP-2-CT**
MMP-9-E	--							
MMP-9-CT	0.319	--						
TIMP-1-E	0.298	0.036	--					
TIMP-1-CT	0.248	0.214	0.372	--				
TIMP-4-CT	0.308	0.624	0.344	0.195	--			
TIMP-4-E	0.326	0.128	0.667	0.248	0.308	--		
TIMP-2-E	0.130	−0.350	0.453	0.276	0.098	0.478	--	
TIMP-2-CT	−0.077	0.395	−0.096	−0.121	0.284	−0.186	0.142	--

Abbreviations: E, epithelium; CT, connective tissue; NT, nerve tissue; PGP 9.5, protein gene product 9.5; MMP, matrix metalloproteinase; TIMP, tissue inhibitor of metalloproteinases. Red means a positive statistically important correlation, blue means statistically important negative correlations.

## Data Availability

The original contributions presented in this study are included in the article. Further inquiries can be directed to the corresponding authors.

## Appendix A

**Table A1:** Semiquantitative assessment of MMP-1, MMP-2, MMP-9, TIMP-1, TIMP-2, and TIMP-4 in EA and control specimens.

(**A**) MMP markers									
**Code**	**Sex**	**Age**	**Part**	**MMP-1 E**	**MMP-1 CT**	**MMP-2 E**	**MMP-2 CT**	**MMP-9 E**	**MMP-9 CT**
9	M	1 d	Prox.	+++	+	++++	0/+	++	+
19	F	1 d	Prox.		+++		0		0
22	M	1 d	Prox.	+	+	+	+	+/++	+
2	F	1 d	Prox.	++	++	++	++	++	0/+
7	M	1 d	Prox.	+/++	0/+	++	+++	+	0
18	F	3 d	Prox.		++		+++		0/+
14	M	4 d	Prox.	+/++	+	++	++	+++	0
15	M	1 m	Prox.		+		+		+/++
5	M	11 m	Prox.	0/+	+/++	+++	++	++	+
4	M	11 m	Prox	+	+	++	++	+	0
20	M	1 d	Dist.	+/++	+++	+	+	++/+++	+/++
21	F	1 d	Dist.	++	+/++	++++	++++	+++	+++
1	F	1 d	Dist.	+/++	0	++	0/+	++	0
12	F	4 d	Dist.		++++		++		++/+++
11	F	4 d	Dist.		+++		++		++
13	M	4 d	Dist.	++	+	0/+	++	+/++	0
10	F	21 d	Dist.		+++/++++		+		+++/++++
6	F	1 d	Dist.	+/++	+++	++	++	+/++	++
8	M	1 d	Dist.	++	+/++	+	+	0/+	0
17	F	1 d	Dist.		+/++	++	+	+	++
3	M	3 d	Dist.	+++	++	+	+	+/++	0/+
16	M	4 m	Dist.		++++		++		+
1k	F	-	C	++/+++	+	++++	++++	0/+	0/+
2k	F	-	C	++/+++	+	+++/++++	+++	+/++	0/+
3k	F	-	C	+++	+	+++	+++	+++	0
4k	M	-	C	++/+++	+	++++	++++	++++	++/+++
5k	M	-	C	++	++/+++	++	++	++/+++	+/++
(**B**) TIMP markers									
Code	**Sex**	**Age**	**Part**	**TIMP-1 E**	**TIMP-1 CT**	**TIMP-4 CT**	**TIMP-4 E**	**TIMP-2 E**	**TIMP-2 CT**
9	M	1 d	Prox.	+	++	0/+	+++	+/++	+/++
19	F	1 d	Prox.	++	++++	++		++++	0/+
22	M	1 d	Prox.	++	++	+++	++	++	++
2	F	1 d	Prox.	+++	+++	++	+++	++	+
7	M	1 d	Prox.	+++	+++	++	++	+++/++++	+/++
18	F	3 d	Prox.		++++	+++			++
14	M	4 d	Prox.	+++	+++	++	+++	+++	0/+
15	M	1 m	Prox.		+++	++	++		+
5	M	11 m	Prox.	++++	++++	+++	++++	+++	+/++
4	M	11 m	Prox	++++	+++	+	++++	+++	0/+
20	M	1 d	Dist.	++	++	+++	+++	+/++	0/+
21	F	1 d	Dist.	++++	++++	++++	++++		0/+
1	F	1 d	Dist.	++++	++++	+	+++	+++	0/+
12	F	4 d	Dist.		+++/++++	++/+++			++++
11	F	4 d	Dist.		+++	++++			+++
13	M	4 d	Dist.	+	++/+++	0/+	+	+++/++++	++/+++
10	F	21 d	Dist.		+++	+++			+++/++++
6	F	1 d	Dist.	+++	++++	++	++	++/+++	+++
8	M	1 d	Dist.	+/++	++	+	+	+	++/+++
17	F	1 d	Dist.	+/++	+++	+++	++	++/+++	++/+++
3	M	3 d	Dist.	+++	++	++	0/+	+	0/+
16	M	4 m	Dist.		+++	+			0/+
1k	F	-	C	+++	++	++	++++	+++	++/+++
2k	F	-	C	++++	++	+++	+++	+++/++++	+++
3k	F	-	C	+++/++++	++	++	+++	++++	+++
4k	M	-	C	++++	+++	+++	++++	++++	+++
5k	M	-	C	++	+++	++	+	0/+	+++

Abbreviations: E, epithelium; CT, connective tissue; C, control; F, female; M, male; Dist., distal segment; Prox., proximal segment; d, day; m, month. Blank cells represent blank source entries.

## Appendix B

**Table A2:** Semiquantitative assessment of PGP 9.5 and motilin in EA and control specimens.

Code	Sex	Age	Part	PGP 9.5 E	PGP 9.5 NT	Motilin E	Motilin CT
9	M	1 d	Prox.	0	++	+++	0
19	F	1 d	Prox.		+++		++
22	M	1 d	Prox.	0/+	+++	0	+++
2	F	1 d	Prox.	0/+	+++	0/+	++
7	M	1 d	Prox.	0/+	+++	++	+++
18	F	3 d	Prox.		+++	0/+	+
14	M	4 d	Prox.	0	+++	+++	++
15	M	1 m	Prox.	0	+++		++++
5	M	11 m	Prox.	0/+	++++	+++	0/+
4	M	11 m	Prox	0	+++	0	0/+
20	M	1 d	Dist.	+	++++	0	+++
21	F	1 d	Dist.	+++	++++	0	+++
1	F	1 d	Dist.	0/+	+++	0	0
12	F	4 d	Dist.		+++		++
11	F	4 d	Dist.	+++	++++	0	++
13	M	4 d	Dist.	0	+++	+	+
10	F	21 d	Dist.	0	+/++	0	+++
6	F	1 d	Dist.	0	+++	0/+	0/+
8	M	1 d	Dist.	0	+++	+	0
17	F	1 d	Dist.	0/+	+++	0	0/+
3	M	3 d	Dist.	0	+++	0	+++
16	M	4 m	Dist.		+		+
1k	F	-	C	0	+++	0	0
2k	F	-	C	0/+	+++	0	0
3k	F	-	C	0	+++	0/+	0
4k	M	-	C	0	++++	0	0
5k	M	-	C	0	++	0	+++

Abbreviations: E, epithelium; CT, connective tissue; NT, nerve tissue; C, control; F, female; M, male; Dist., distal segment; Prox., proximal segment; d, day; m, month. Blank cells represent blank source entries.

## References

[1] Que J., Choi M., Ziel J.W., Klingensmith J., Hogan B.L.M. (2006). Morphogenesis of the trachea and esophagus: Current players and new roles for noggin and Bmps. Differentiation.

[2] Spitz L. (2007). Oesophageal atresia. Orphanet J. Rare Dis..

[3] Shaw-Smith C. (2006). Oesophageal atresia, tracheo-oesophageal fistula, and the VACTERL association. J. Med. Genet..

[4] Pedersen R.N., Calzolari E., Husby S., Garne E., EUROCAT Working Group (2012). Oesophageal atresia: Prevalence, prenatal diagnosis and associated anomalies in 23 European regions. Arch. Dis. Child..

[5] Nassar N., Leoncini E., Amar E., Arteaga-Vázquez J., Bakker M.K., Bower C., Canfield M.A., Castilla E.E., Cocchi G., Correa A. (2012). Prevalence of esophageal atresia among 18 international birth defects surveillance programs. Birth Defects Res. Part A Clin. Mol. Teratol..

[6] Comella A., Tan T., Hutson J.M., Omari T.I., Teague W.J., Nataraja R.M., King S.K. (2021). Esophageal morbidity in patients following repair of esophageal atresia: A systematic review. J. Pediatr. Surg..

[7] Gottrand M., Michaud L., Sfeir R., Gottrand F. (2016). Motility, digestive and nutritional problems in esophageal atresia. Paediatr. Respir. Rev..

[8] Faure C., Righini Grunder F. (2017). Dysmotility in Esophageal Atresia: Pathophysiology, Characterization, and Treatment. Front. Pediatr..

[9] Maestri F., Morandi A., Ichino M., Fava G., Cavallaro G., Leva E., Macchini F. (2022). Endoscopic Surveillance of Esophageal Atresia Population according to ESPGHAN-NASPGHAN 2016 Guidelines: Incidence of Eosinophilic Esophagitis and New Histological Findings. Biomedicines.

[10] Krishnan U., Mousa H., Dall’Oglio L., Homaira N., Rosen R., Faure C., Gottrand F. (2016). ESPGHAN-NASPGHAN Guidelines for the Evaluation and Treatment of Gastrointestinal and Nutritional Complications in Children With Esophageal Atresia-Tracheoesophageal Fistula. J. Pediatr. Gastroenterol. Nutr..

[11] Pinheiro P.F.M., Simoes e Silva A.C., Pereira R.M. (2012). Current knowledge on esophageal atresia. World J. Gastroenterol..

[12] de Jong E.M., Felix J.F., de Klein A., Tibboel D. (2010). Etiology of esophageal atresia and tracheoesophageal fistula: Mind the Gap. Curr. Gastroenterol. Rep..

[13] Sistonen S., Koivusalo A., Nieminen U., Lindahl H., Lohi J., Kero M., Kärkkäinen P.A., Färkkilä M.A., Sarna S., Rintala R.J. (2010). Esophageal morbidity and function in adults with repaired esophageal atresia with tracheoesophageal fistula: A population-based long-term follow-up. Ann. Surg..

[14] Lemoine C., Aspirot A., Le Henaff G., Piloquet H., Levesque D., Faure C. (2013). Characterization of esophageal motility following esophageal atresia repair using high-resolution esophageal manometry. J. Pediatr. Gastroenterol. Nutr..

[15] van der Zee D.C., Bagolan P., Faure C., Gottrand F., Jennings R., Laberge J.M., Ferro M.H.M., Parmentier B., Sfeir R., Teague W. (2017). Position Paper of INoEA Working Group on Long-Gap Esophageal Atresia: For Better Care. Front. Pediatr..

[16] Mahoney L., Rosen R. (2017). Feeding Problems and Their Underlying Mechanisms in the Esophageal Atresia-Tracheoesophageal Fistula Patient. Front. Pediatr..

[17] Koumbourlis A.C., Belessis Y., Cataletto M., Cutrera R., DeBoer E.M., Kazachkov M., Laberge S., Popler J., Porcaro F., Kovesi T. (2020). Care Recommendations for the Respiratory Complications of Esophageal Atresia-Tracheoesophageal Fistula. Pediatr. Pulmonol..

[18] Lal D.R., Gadepalli S.K., Downard C.D., Ostlie D.J., Minneci P.C., Swedler R.M., Chelius T., Cassidy L., Rapp C.T., Deans K.J. (2017). Perioperative management and outcomes of esophageal atresia and tracheoesophageal fistula. J. Pediatr. Surg..

[19] Baird R., Lal D.R., Ricca R.L., Diefenbach K.A., Downard C.D., Shelton J., Sømme S., Grabowski J., A Oyetunji T., Williams R.F. (2019). Management of long gap esophageal atresia: A systematic review and evidence-based guidelines from the APSA Outcomes and Evidence Based Practice Committee. J. Pediatr. Surg..

[20] Pilmane M., Ozolina A., Abola Z., Petersons A. (2011). Growth factors, their receptors, neuropeptide-containing innervation and matrix metalloproteinases in the proximal and distal esophagus in children with esophageal atresia. Medicina.

[21] Aspirot A., Faure C. (2013). Esophageal dysmotility: Characterization and pathophysiology. Dis. Esophagus.

[22] Tovar J.A., Diez Pardo J.A., Murcia J., Prieto G., Molina M., Polanco I. (1995). Ambulatory 24-hour manometric and pH-metric evidence of permanent impairment of clearance capacity in patients with esophageal atresia. J. Pediatr. Surg..

[23] Lemoine C., Aspirot A., Morris M., Faure C. (2015). Esophageal dysmotility is present before surgery in isolated tracheoesophageal fistula. J. Pediatr. Gastroenterol. Nutr..

[24] Dutta H.K., Mathur M., Bhatnagar V. (2000). A histopathological study of esophageal atresia and tracheoesophageal fistula. J. Pediatr. Surg..

[25] Boleken M., Demirbilek S., Kirimiloglu H., Kanmaz T., Yucesan S. (2007). Reduced neuronal innervation in the distal end of the proximal esophageal atretic segment in cases of esophageal atresia with distal tracheoesophageal fistula. World J. Surg..

[26] Li K., Zheng S., Xiao X., Wang Q., Zhou Y., Chen L. (2007). The structural characteristics and expression of neuropeptides in the esophagus of patients with congenital esophageal atresia and tracheoesophageal fistula. J. Pediatr. Surg..

[27] Qi B.Q., Uemura S., Farmer P., Myers N.A., Hutson J.M. (1999). Intrinsic innervation of the oesophagus in fetal rats with oesophageal atresia. Pediatr. Surg. Int..

[28] Qi B.Q., Merei J., Farmer P., Hasthorpe S., A Myers N., Beasley S.W., Hutson J.M. (1997). The vagus and recurrent laryngeal nerves in the rodent experimental model of esophageal atresia. J. Pediatr. Surg..

[29] Nakazato Y., Wells T.R., Landing B.H. (1986). Abnormal tracheal innervation in patients with esophageal atresia and tracheoesophageal fistula: Study of the intrinsic tracheal nerve plexuses by a microdissection technique. J. Pediatr. Surg..

[30] Thompson R.J., Doran J.F., Jackson P., Dhillon A.P., Rode J. (1983). PGP 9.5-a new marker for vertebrate neurons and neuroendocrine cells. Brain Res..

[31] Wilkinson K.D., Lee K.M., Deshpande S., Duerksen-Hughes P., Boss J.M., Pohl J. (1989). The neuron-specific protein PGP 9.5 is a ubiquitin carboxyl-terminal hydrolase. Science.

[32] Wilson P.O., Barber P.C., Hamid Q.A., Power B., Dhillon A., Rode J., Day I., Thompson R., Polak J. (1988). The immunolocalization of protein gene product 9.5 using rabbit polyclonal and mouse monoclonal antibodies. Br. J. Exp. Pathol..

[33] Day I.N.M., Thompson R.J. (2010). UCHL1 (PGP 9.5): Neuronal biomarker and ubiquitin system protein. Prog. Neurobiol..

[34] Kitazawa T., Kaiya H. (2021). Motilin Comparative Study: Structure, Distribution, Receptors, and Gastrointestinal Motility. Front. Endocrinol..

[35] Deloose E., Verbeure W., Depoortere I., Tack J. (2019). Motilin: From gastric motility stimulation to hunger signalling. Nat. Rev. Endocrinol..

[36] Chaussade S., Michopoulos S., Sogni P., Guerre J., Couturier D. (1994). Motilin agonist erythromycin increases human lower esophageal sphincter pressure by stimulation of cholinergic nerves. Dig. Dis. Sci..

[37] Coulie B., Tack J., Peeters T., Janssens J. (2010). Effect of atilmotin, a motilin receptor agonist, on esophageal, lower esophageal sphincter and gastric contractility. Neurogastroenterol. Motil..

[38] Chrysos E., Tzovaras G., Epanomeritakis E., Tsiaoussis J., Vrachasotakis N., Vassilakis J.S., Xynos E. (2001). Erythromycin enhances oesophageal motility in patients with gastro-oesophageal reflux. ANZ J. Surg..

[39] Cabral-Pacheco G.A., Garza-Veloz I., Castruita-De Leon M.C., Ramirez-Acuña J.M., Perez-Romero B.A., Guerrero-Rodriguez J.F., Martinez-Avila N., Martinez-Fierro M.L. (2020). The roles of matrix metalloproteinases and their inhibitors in human diseases. Int. J. Mol. Sci..

[40] Caley M.P., Martins V.L.C., O’Toole E.A. (2015). Metalloproteinases and Wound Healing. Adv. Wound Care.

[41] Nagase H., Visse R., Murphy G. (2006). Structure and function of matrix metalloproteinases and TIMPs. Cardiovasc. Res..

[42] Murphy G. (2011). Tissue inhibitors of metalloproteinases. Genome Biol..

[43] Arpino V., Brock M., Gill S.E. (2015). The role of TIMPs in regulation of extracellular matrix proteolysis. Matrix Biol..

[44] Stamenkovic I. (2003). Extracellular matrix remodelling: The role of matrix metalloproteinases. J. Pathol..

[45] Brew K., Nagase H. (2010). The tissue inhibitors of metalloproteinases (TIMPs): An ancient family with structural and functional diversity. Biochim. Biophys. Acta.

[46] Melendez-Zajgla J., Del Pozo L., Ceballos G., Maldonado V. (2008). Tissue inhibitor of metalloproteinases-4: The road less traveled. Mol. Cancer.

[47] Guo Y.H., Gao W., Li Q., Li P.F., Yao P.Y., Chen K. (2004). Over-expression of TIMP-4 inhibits activation of MMP-2 and induces apoptosis in rat vascular smooth muscle cells. Life Sci..

[48] Zhang J., Zhao Y.G., Cao Y.J., Sang Q.X., Duan E.K. (2003). Expression and implications of tissue inhibitor of metalloproteinases-4 in mouse embryo. Mol. Hum. Reprod..

[49] Ozola L., Pilmane M. (2024). Characterization of Tissue Immunity Defense Factors of the Lip in Primary Dentition Children with Bilateral Cleft Lip Palate. J. Pers. Med..

[50] Barton B., Peat J. (2014). Medical Statistics: A Guide to SPSS, Data Analysis and Critical Appraisal.

[51] Al-Shraim M.M., Eid R.A., Musalam A.O., Radad K., Ibrahim A.H.M., Malki T.A. (2015). Ultrastructural changes of the smooth muscle in esophageal atresia. Ultrastruct. Pathol..

[52] Pinzon-Guzman C., Sangadala S., Riera K.M., Popova E.Y., Manning E., Huh W.J., Alexander M.S., Shelton J.S., Boden S.D., Goldenring J.R. (2020). Noggin regulates foregut progenitor cell programming, and misexpression leads to esophageal atresia. J. Clin. Investig..

[53] Broad J., Mukherjee S., Samadi M., Martin J.E., Dukes G.E., Sanger G.J. (2012). Regional- and agonist-dependent facilitation of human neurogastrointestinal functions by motilin receptor agonists. Br. J. Pharmacol..

[54] Nishikubo T., Yamakawa A., Kamitsuji H., Nakajima M., Takahashi Y., Nakayama M., Kitajima H., Fujimura M. (2005). Identification of the motilin cells in duodenal epithelium of premature infants. Pediatr. Int..

[55] Leduque P., Paulin C., Chayvialle J.A., Dubois P.M. (1981). Immunocytological evidence of motilin- and secretin-containing cells in the human fetal gastro-entero-pancreatic system. Cell Tissue Res..

[56] Bryant M.G., Buchan A.M., Gregor M., Ghatei M.A., Polak J.M., Bloom S.R. (1982). Development of intestinal regulatory peptides in the human fetus. Gastroenterology.

[57] Vaivads M., Akota I., Pilmane M. (2021). Cleft Candidate Genes and Their Products in Human Unilateral Cleft Lip Tissue. Diseases.

[58] Vitenberga Z., Pilmane M., Babjoniseva A. (2019). The evaluation of inflammatory, anti-inflammatory and regulatory factors contributing to the pathogenesis of COPD in airways. Pathol. Res. Pract..

[59] Vaivads M., Akota I., Pilmane M. (2023). Characterization of SHH, SOX3, WNT3A and WNT9B Proteins in Human Non-Syndromic Cleft Lip and Palate Tissue. Dent. J..

[60] Denisova A., Pilmane M., Engelis A., Petersons A. (2021). Gallbladder Interleukins in Children with Calculous Cholecystitis. Pediatr. Rep..

[61] Taylor C.R., Levenson R.M. (2006). Quantification of immunohistochemistry-issues concerning methods, utility and semiquantitative assessment II. Histopathology.

[62] Rone A.E., Junga A., Akota I., Pilmane M. (2026). Non-Syndromic or Anomaly-Associated Genes (MYH3, GREM1, IRF6) and Their Proteins in Unilateral Right Cleft Tissue. Int. J. Mol. Sci..

[63] Akoglu H. (2018). User’s guide to correlation coefficients. Turk. J. Emerg. Med..

[64] Apte S.S., Parks W.C. (2015). Metalloproteinases: A parade of functions in matrix biology and an outlook for the future. Matrix Biol..

[65] Moliere S., Jaulin A., Tomasetto C.L., Dali-Youcef N. (2023). Roles of Matrix Metalloproteinases and Their Natural Inhibitors in Metabolism: Insights into Health and Disease. Int. J. Mol. Sci..

[66] Andl C.D., Mizushima T., Oyama K., Bowser M., Nakagawa H., Rustgi A.K. (2004). EGFR-induced cell migration is mediated predominantly by the JAK-STAT pathway in primary esophageal keratinocytes. Am. J. Physiol. Liver Physiol..

[67] Rivera S., Tremblay E., Timsit S., Canals O., Ben-Ari Y., Khrestchatisky M. (1997). Tissue inhibitor of metalloproteinases-1 (TIMP-1) is differentially induced in neurons and astrocytes after seizures: Evidence for developmental, immediate early gene, and lesion response. J. Neurosci..

[68] Jaworski D.M., Pérez-Martínez L. (2006). Tissue inhibitor of metalloproteinase-2 (TIMP-2) expression is regulated by multiple neural differentiation signals. J. Neurochem..

[69] Young D.A., Phillips B.W., Lundy C., Nuttall R.K., Hogan A., Schultz G.A., Leco K.J., Clark I.M., Edwards D.R. (2002). Identification of an initiator-like element essential for the expression of the tissue inhibitor of metalloproteinases-4 (Timp-4) gene. Biochem. J..

[70] Wang Z., Juttermann R., Soloway P.D. (2000). TIMP-2 is required for efficient activation of proMMP-2 in vivo. J. Biol. Chem..

[71] Stetler-Stevenson W.G., Seo D.W. (2005). TIMP-2: An endogenous inhibitor of angiogenesis. Trends Mol. Med..

